# Experimental induction of necrotic enteritis with or without predisposing factors using *netB* positive *Clostridium perfringens* strains

**DOI:** 10.1186/s13099-021-00463-z

**Published:** 2021-11-17

**Authors:** Mudassar Mohiuddin, Weikang Yuan, Zhongfeng Song, Shenquan Liao, Nanshan Qi, Juan Li, Minna Lv, Caiyan Wu, Xuhui Lin, Junjing Hu, Haiming Cai, Mingfei Sun

**Affiliations:** 1 Key Laboratory of Livestock Disease Prevention of Guangdong Province, Maoming Branch, Guangdong Laboratory for Lingnan Modern Agriculture, Scientific Observation and Experiment Station of Veterinary Drugs and Diagnostic Techniques of Guangdong Province, Ministry of Agriculture, Institute of Animal Health, Guangdong Academy of Agricultural Sciences, Guangzhou, 510640 China; 2 Department of Microbiology, Faculty of Veterinary and Animal Sciences, The Islamia University of Bahawalpur, Bahawalpur, 63100 Pakistan

**Keywords:** Necrotic enteritis, *C. perfringens*, *Eimeria necatrix*, Fish meal, Intestinal lesions

## Abstract

**Background:**

Poultry necrotic enteritis (NE) is an economically important disease caused by *C. perfringens*. The disease causing ability of this bacterium is linked with the production of a wide variety of toxins. Among them, necrotic enteritis B-like (*NetB*) toxin is reported to be involved in the pathogenesis of NE; in addition there is some circumstantial evidence that *tpeL* toxin may enhance virulence, but this is yet to be definitely shown. The situation becomes more complicated in the presence of a number of predisposing factors like co-infection with coccidia, type of diet and use of high protein diet. These co-factors alter the intestinal environment, thereby favoring the production of more toxins, leading to a more severe disease. The objective of this study was to develop a successful animal model that would induce clinical signs and lesions of NE using *C. perfringens* type G strains obtained from field outbreaks. A separate trial was simultaneously considered to establish the role of dietary factor with coccidial co-infection in NE.

**Results:**

The results have shown that use of *net-B* positive *C. perfringens* without predisposing factors induce moderate to severe NE (Av. Lesion score 1.79 ± 1.50). In a separate trial, addition of fish meal to a feed of *C. perfringens* challenged birds produced higher number of NE cases (Av. Lesion score 2.17 ± 1.28). However, use of less virulent *E. necatrix* strain along with fish meal in conjunction with *net-B* positive strain did not alter the severity of NE lesions in specific pathogen free chicken (Av. Lesion score 2.21 ± 1.13).

**Conclusions:**

This study suggests that virulent *C. perfringens* type G strains can induce NE lesions in the absence of other predisposing factors. Birds in the clostridia challenged group showed moderate to severe NE lesions. Use of less virulent coccidia strain contributed to a lesser extent in increasing the severity of disease. Maize based diet along with fishmeal (1:1) increased the severity of lesions but statistically it was non-significant. The NE lesions in all experimental groups were found to be present more frequently in the duodenum. In this way, this study provided an effective model for in vivo production of NE in poultry birds.

## Introduction

Necrotic enteritis (NE), a reemerging threat to the poultry industry, has been well controlled for many years by the use of in-feed antibiotics and antimicrobial growth promoters (AGPs) [1]. These were the most effective strategies, until a few years ago, to overcome huge losses to what has been now called a $6 billion disease [2]. This spike in the incidence of NE has resulted from the ban in many countries on the use of antibiotics and AGPs [3]. The situation arises because of the rising concerns about spreading of antimicrobial resistance from animals to human. On the other hand, increasing world population, demands more availability of quality protein and the poultry sector is one of the richest source to fulfil those protein needs of humans. Therefore, finding alternative ways to control NE infection are important to overcome food scarcity challenges and ensuring health safety.

The disease is caused by *Clostridium perfringens*, a gram positive, anaerobic, endospore forming bacterium [4, 5]. Proliferation of this microorganism in the intestinal tract leads to toxin production, thereby inducing characteristic NE lesions and clinical signs of enteritis. According to the new toxinotyping scheme, *C. perfringens* type G is main causative agent of clostridial enteritis in chicken. The type G strains have the *netB* gene, encoding necrotic enteritis B-like (*NetB*) toxin, and also produce alpha toxin. In addition, type G may also contain other toxins including *cpb2* and *tpeL* [6, 7]. There have been rising concerns about the extent of involvement of *netB* toxin in causing NE in the absence of predisposing factors. This is because, *netB* gene has also been isolated from healthy chicken [8]. Previously, some researchers do believe that type A alone producing alpha toxin, can cause NE infection on the basis of animal model studies [9]. This was later on questioned by many researchers, when type G mutant strain lacking alpha toxin induced NE in chicken. The role of predisposing factors is however critical in increasing the severity of disease and is important to understand [10]. This demands inducing NE in a controlled manner with and without other factors to clearly underline the main cause of this disease and effectively overcoming huge economic losses to poultry sector.

Clinical NE has been reproduced experimentally with or without other predisposing factors; many of these have been unveiled and incorporated successfully in various animal model studies from time to time. Reproducibility is a challenging task, keeping in view the multifactorial nature of disease and intervention strategies applied. The reproduction and severity of NE alone by using clostridial bacteria depends on *C. perfringens* type, presence of additional toxins, protocol for enrichment of pure culture and the route, timing, dose and frequency of the bacterial challenge [11–13]. The predominant predisposing factors which are known to play an important role in induction and severity of NE were also targeted. These included using high protein diet i.e., fish meal with a maize based diet (1:1) and co-infection with coccidia [11, 14–16]. The freshly isolated and confirmed *C. perfringens* type G strains obtained from field outbreak were used for induction model. These multi-experimental models successfully yielded NE gross lesions in the majority of challenged birds with as well as without other predisposing factors.

## Results

### Trial 1

In Trial 1, the average NE lesion scores for CP(FJ6)(C8-1) and E.n(G) groups were 1.79 ± 1.50 and 1.08 ± 1.53 respectively. Their results were significantly different from those of the control group (0.13 ± 0.34) i.e., (P < 0.001). The ability of two local strains; CP(FJ6) and (C8-1) to produce NE was examined without dietary and coccidia predisposing factor in this trial. Lesions were found to be present on each sampling day (17, 19 and 23). The lesions in the E.n group were less severe as compared to the Clostridia group and developed subclinical NE in 41.7% birds only. The small intestine was distended and there was thinning of walls. Group 3 which was the control group did not develop any signs of NE (Table 1).

**Table 1:** Frequency of NE lesions in chicks challenged with various *C. perfringens* strains and other predisposing factors

Trial	Group	Dietary factor^d^	NE lesion score							NE case^e^	NE incidence (%)
			0	1	2	3	4	Sub total	Mean		
1	CP(FJ6)(C8-1)^a^	−	6	6	4	3	5	24	1.79*	12	50
	E.n(G)^b^	−	15	0	4	2	3	24	1.08*	09	41.7
	CTL^c^	−	20	3	0	0	0	23^f^	0.13	0	0
2	CP(FJ6)(C8-1)	+	2	6	6	5	5	24	2.17*	16	66.7
	E.n(G)	+	9	4	7	1	2	23^f^	1.22*	10	43.5
	CP(FJ6) (C8-1) + E.n(G)	+	2	5	6	9	2	24	2.21*	17	70.8
	CTL	+	19	5	0	0	0	24	0.21	0	0

^a^CP(FJ6) (C8-1): *Clostridium perfringens* type G strain (FJ6)(C8-1)

^b^E.n(G): *Eimeria necatrix* Guangdong strain

^c^CTL: control group

^d^Dietary factor: maize-based diet containing 60% fishmeal (1:1)

^e^NE case is defined by lesion score reaching 2 or above

^f^01 chicks died as early mortality in the group CTL and *Eimeria*, dissimilar letters indicate a significant difference compared with CTL group at 95% confidence level (independent t-test)

*Means highly significant (*P* < 0.01)

### Trial 2

In the Trial 2, fish meal was added to the maize based diet in the ratio of (1:1) and offered to the chicks from 8th day onward. The NE lesion scores for the CP(FJ6)(C8-1), E.n(G) and CP(FJ6)(C8-1) + E.n(G) groups were 2.17 ± 1.28, 1.22 ± 1.29, 2.21 ± 1.13, respectively. These results were highly significant as compared to the CTL group (0.21 ± 0.41). No significant difference was observed in the NE lesions for the CP(FJ6)(C8-1) and CP(FJ6)(C8-1) + E.n(G) groups (Table 1). Lesions similar to sub-clinical NE were observed in this experiment. Moreover, the characteristic macroscopic lesions were observed at each sampling time i.e., day 19, 21 and 23. About 66.7% of the infected chicks in the CP(FJ6)(C8-1) group were having NE lesion score of 2 or more than 2 having small intestine not only distended due to gas production but also thin walled. Most lesions were focal necrosis and no mortality was observed in the infected chicks. Most of the birds having sub-clinical NE showed small necrotic foci (6 or more in number), distributed in the proximal half of the small intestine. Some of the lesions were extensive and presented confluent picture of that segment. In case of CP(FJ6)(C8-1) group, lesions were most prominent in the duodenum, which in some cases extended to jejunum and ileum also. Moreover, the lesions observed in case of E.n(G) + dietary factor group had non-significant difference with the lesions of NE seen in case of trial 1 for E.n(G) group alone without dietary factor.

### *Clostridium perfringens* strains (FJ6 and C8-1)

**Fig. 1:**
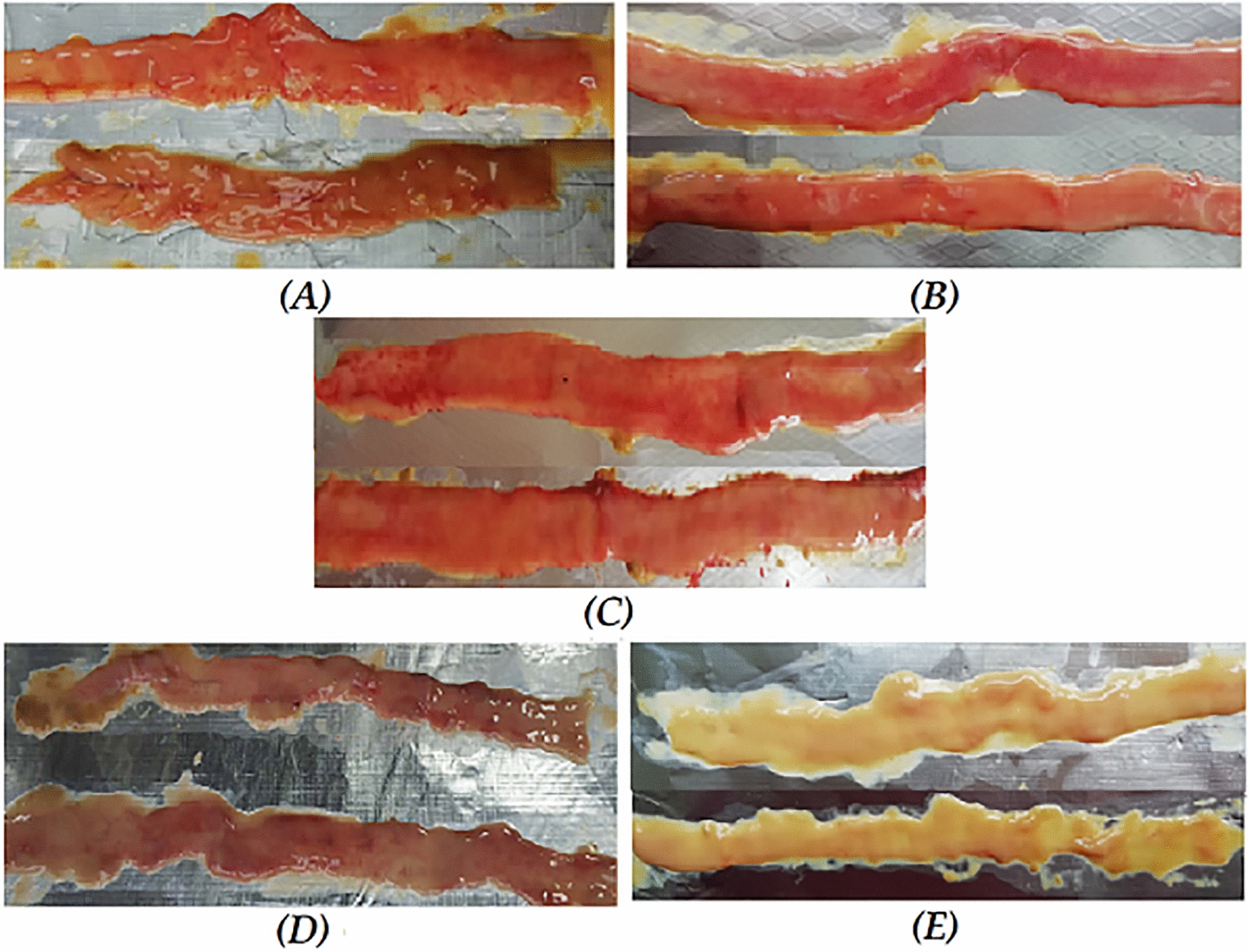
Gross typical lesions of experimentally induced Necrotic enteritis in duodenum and jejunum (**A**) CP(FJ6) (**B**) CP(FJ6) + dietary factor (**C**) CP(FJ6) + dietary factor + E.n(G) (**D**) *Eimeria necatrix* (G) (**E**) control

Induction of NE using FJ6 and C8-1 strains was carried out in specific pathogen free (spf) chicks using maize based diet. In the absence of other predisposing factors; FJ6 strain produce NE lesions in 83.3% and C8-1 strain successfully induce NE in 66.7% of birds and in both cases, the majority of the lesions were present in duodenum. High protein diet along with co-infection with coccidia (*Eimeria necatrix* strain) increased the lesion score by 87.5% and 100% for FJ6 and C8-1 strains respectively (Table 2). The increase in the production of lesions in case of C8-1 strain was prominent as compared to FJ6 strain. Overall, the predisposing factors did increase the number and severity of lesions to some extent in small intestine (Fig. 1).

**Table 2:** Response of chicks to challenge with various *C. perfringens* strains or co-infecting with *E. necatrix*

*C. perfringens* strain	Co-infection^c^	Dietary factor^d^	Birds with gross lesions/total birds (%)	Birds with lesions in jejunum (%)	Birds with lesions in duodenum (%)
CP(FJ6)^a^	−	−	10/12 (83.3%)	4/12 (33.3%)	10/12 (83.3%)
	−	+	11/12 (91.7%)	7/12 (58.3%)	11/12 (91.7%)
	+	+	7/8 (87.5%)	6/8 (75%)	7/8 (87.5%)
CP(C8-1)^b^	−	−	8/12 (66.7%)	2/12 (16.7%)	8/12 (66.7%)
	−	+	11/12 (91.7%)	7/12 (58.3%)	11/12 (91.7%)
	+	+	8/8 (100%)	2/8 (25%)	8/8 (100%)

^a^CP(FJ6) and ^b^(C8-1): *C. perfringens* type G strains

^c^Co-infection: group co-infected with *Eimeria necatrix* Guangdong strain

^d^Dietary factor: maize-based diet containing 60% fishmeal (1:1)

### *Eimeria necatrix* (E.n) (Guangdong) strain

The E. necatrix (E.n) Guangdong strain identified and characterized by our lab also induces NE in birds, however there was no significant difference in the lesion score of birds treated with E.n(G) alone (1.08 ± 1.53) or E.n(G) along with fish meal (1.22 ± 1.29). The strain alone resulted in coccidia infection in 41.7% while along with high protein diet produced 43.5% incidence rate in chicken (Table 2).

## Discussion

Several studies have been carried out to understand the explicit role of *C. perfringens* in causing NE. It was initially found that *C. perfringens* type A having alpha toxin gene is responsible for causing this disease in poultry [9]. Later on, with the discovery of new toxin in 2008, it was found that the presence of *netB* toxin is essential [6] and the toxinotype having *netB* toxin gene in addition to alpha gene was named as *C. perfringens* type G in 2018 (which may or may not be having additional toxin genes) [5]. Some studies still claim that type A alone can also cause NE in poultry [9] while many others insisted the presence of *netB* toxin gene as a key factor in causing the disease [17]. Interestingly, *C. perfringens* type G strains sometimes can also be isolated from healthy chicken [8, 18]. In this perspective, researchers have found that other predisposing factors are of key importance, which alters the intestinal environment and favors more toxin production leading to moderate to severe intestinal lesions [10]. Besides several other critical factors, coinfection with coccidia, high protein diet and use of wheat as a dietary source are considered to play a vital role in developing NE infection [19, 20]. This study established the fact that *C. perfringens* type G alone is sufficient to cause NE while other factors, which are critical and play an important role in determining/enhancing the virulence of *C. perfringens* toxinotypes were not able to significantly enhance the NE lesion score in the chicken [17]. The strains used in this study were freshly isolated from field NE outbreak and also grown in enriched media before preparation of inoculum for use according to the set protocol. Fresh 18–20 h culture of *C. perfringens* was used for oral administration to chicks.

In this study, the role of *C. perfringens* was studied in causing NE with or without other predisposing factors. The main focus of this animal model experimentation was to find out the influence of *netB* positive strains, which was successfully established. The type G strains used were confirmed as positive for *netB* gene by Sanger dideoxy sequencing method and these NE positive isolates were not positive for other toxins including *cpb2*, *tpeL*, and *cpe* (data not shown). CP(FJ6) and (C8-1) strains were recently isolated field strains with no in vivo and limited in vitro passages, and were observed to produce lesions in more than 50% of challenged chicks. Some other studies also established the fact that only *netB* strain can reproduce consistent levels of disease [17, 21–23]. It is also noted in this experimental model, that both *C. perfringens* strains produced severe lesions and more number of foci in the duodenum as compared to jejunum and ileum [24]. Some other experimental models reported more lesions in the jejunum part of small intestine [9].

In the presence of dietary factor, there was non-significant increase in the severity and lesion score in the challenged birds. One reason for this non-significant increase might be the use of maize based diet, which according to different studies had a lesser impact on NE lesion score than wheat based or other diets, which can somehow delay the retention of diet in the intestine, thereby facilitate the multiplication of microflora at a higher rate. *Eimeria necatrix* was used alone in this study to cause NE which resulted in significant lesion score in the chicken as compared to the control group however this strain did not enhance the severity of lesions significantly, when used in combination with *C. perfringens* strains and dietary factor.

## Conclusions

The present study has established the fact that type G alone can be a major contributor in causing NE. However, this effect might be more severe in the presence of other toxins, which will be considered in the further studies. Three to four doses of CP(FJ6) and CP(C8-1) produced lesions in more than 50% of birds. *Eimeria necatrix* strain also produced moderate coccidial infection in the chicks, however; there was non-significant increase in severity of disease when used along with CP strains and/or high protein diet. In this way, this animal model allows the differentiation of virulent strains isolated from local commercial poultry farms and will be useful for understanding the pathogenesis and immunity against these infections in future studies.

## Materials and methods

### Chicks, housing facility and diets

One day old specific pathogen free (spf) chicks (n = 168) were received from commercial hatchery of Guangdong province, China. The chicks were randomly divided into seven groups and kept in separate metallic cages having fresh litter as bedding for first few days. The birds were offered ad-libitum access to feed and fresh water. No additives i.e., antibiotics and/or anticoccidials were added to the feed. For Trial 1, maize based diet having minimal 21% crude protein was fed to the chicken throughout the experiment. In Trial 2, the same maize based diet was offered for first 07 days and from day 08 onward till the end of experiment, the diet was mixed with fish meal having 60% crude protein in the ratio of 1:1.

### Experimental design

The experiment was carried out using NetB positive *C. perfringens* strains with or without other predisposing factors. The induction of necrotic enteritis (NE) was evaluated on the basis of clinical signs and lesion score of 2 or more in the intestine including duodenum, jejunum, ileum and caecum. The two trials were carried out at the same time. The information concerning experimental designs is presented in the Table 3.

**Table 3:** Trail designs

Trial	N^a^	Groups	Dietary factor^e^	Coccidia challenge	*C. perfringens* challenge	Sampling day
1	24	G1(CP(FJ6) (C8-1))^b^	−	−	Day 14, 15, 16	Day 17(D1)^g^, 19(D3)^h^, 23
	24	G2(E.n(G))^c^	−	Day 09	−	
	24	G3(CTL)^d^	−	−	−	
2	24	G4(CP(FJ6) (C8-1))	+^f^	−	Day 15,16,17,18	Day 19 (D1), 21(D3), 23
	24	G5(E.n(G))	+	Day 10	−	
	24	G6(CP(FJ6) (C8-1) + E.n(G))	+	Day 10	Day 15,16,17,18	
	24	G7(CTL)	+	−	−	

^a^N: number of chickens

^b^CP(FJ6) and (C8-1): *C. perfringens* type G strain (FJ6), (C8-1)

^c^E.n(G)*Eimeria necatrix* Guangdong strain

^d^CTL: control group

^e^Dietary factor: maize-based diet containing 60% fishmeal (1:1)

^f^Day 08 onward till the end of trial

^g^D1: 1 day after the last challenge of *C. perfringens* type G strain

^h^D3: 3 days after the last challenge of *C. perfringens* type G strain

In trial 1, a maize based diet was given to chicks kept in three separate groups. Each group was having 24 chickens. Moreover, group 1 was subdivided into two subgroups (12 chicks each); each subgroup was given challenge with different *C. perfringens* strain. For experimental induction of NE, birds in the group 1 were given 3 ml (2.5 × 10^8^ cfu/ml) of freshly prepared *C. perfringens* culture starting from day 14, for 3 consecutive days. The birds in the group 2 were challenged with coccidia (*Eimeria necatrix*) at the dose rate of 5 × 10^3^ oocysts/ml on day 9. Group 3 was control group, which was given 3 ml normal saline orally. Eight birds from each group were humanely euthanized on each sampling day i.e., 17, 19 and 23 to check NE lesion score.

In trial 2, dietary factor (fish meal) was added to the maize based diet with or without co-infection with coccidia (*Eimeria necatrix*). Group 4 in this trial was given fish meal from day 8 onward and given clostridia challenge on day 15, 16, 17 and 18. This group also comprised two subgroups (12 chicks each) (each subgroup was exposed to different strain using same amount of fresh inocula). Group 5 was given dietary factor and coccidia co-infection only. *E. necatrix* was offered at the dosage 5 × 10^3^ oocysts/ml. Group 6 was given exposure to all predisposing factors i.e., fish meal diet from day 8 onward, *E. necatrix* on day 10, *C. perfringens* inoculum 3 ml containing 2.5 × 10^8^ cfu/ml orally on day 15, 16, 17 and 18. Both Clostridia strains were used in group 6 thereby dividing it into two subgroups. Sampling for trial 2 i.e., group 4, 5, 6 and 7 was done at day 19, 21 and 23 by euthanizing 8 birds from each group each sampling day.

Both trials were reviewed and approved by the ethical review committee of Institute of Animal Health, Guangdong Academy of Agricultural Sciences (No. B8201908).

### Inoculum preparation

The *netB* positive strains used in this study were identified and confirmed by sequencing of PCR product (data not shown). These were clinical isolates collected from suspected NE cases. The isolates were identified as *Clostridium perfringens* toxinotype G which were negative for *tpeL* and *cpb2* by qpcr. The strain was freshly isolated and haven’t yet used in any experimental study before this trial.

To refresh the bacterial culture, pure 2–3 colonies from perfringens agar for each strain were inoculated in 10 ml cooked meat broth and incubated anaerobically in a shaker incubator at 37 °C for 18–24 h. The culture was transferred to BHI broth and subjected to the same growth conditions in ratio of 1:10. This stock culture was used to inoculate 1 litre BHI media. After 18 h, the cells in the broth culture were calculated using spectrophotometer. The OD was measured and the cell number was calculated to make final concentration to 2.5 × 10^8^ cfu/ml. *Eimeria necatrix* was chosen to predispose the chicken to coccidia infection. The strain used was *E. necatrix* Guangdong strain isolated from field outbreak. The dosage 5 × 10^3^ oocysts/ml was used as shown in other studies for successfully inducing NE.

### Lesion scoring

The gross intestinal lesion score (duodenum to ileum) for each bird was noted on the score sheet. The lesion scores ranged from 0 (no lesions grossly), 1 (congestion of intestinal mucosa), 2 (1 to 5 foci), 3 (6 to 15 foci) and 4 (16 or more foci) as described by Prescott et al. [25–27]. In order to avoid biasedness, two persons at the same time observed the intestine and noted the lesion score on all sampling days. The lesion score of 2 or more than 2 was considered as NE positive. The lesion scores of duodenum, jejunum and ileum were noted, however the highest score in each chicken for any segment was considered as the final NE lesion score. For coccidia infection, whole small intestine including caecum was examined for each chicken and scored.

### Statistical analysis

Statistical analysis was performed by IBM SPSS Statistics Base 19.0 software. Significant differences in NE lesion scores between groups were calculated by independent T-test. Fisher’s exact test was used to compare the differences of NE incidence levels between various groups. P < 0.05 was set as the statistically significant difference.

## Data Availability

Not applicable.

## References

[1] Liu D, Guo Y, Wang Z, Yuan J (2010). Exogenous lysozyme influences *Clostridium perfringens* colonization and intestinal barrier function in broiler chickens. Avian Pathol.

[2] Wade B, Keyburn A (2015). The true cost of necrotic enteritis. World Poult.

[3] Gaucher M, Quessy S, Letellier A, Arsenault J, Boulianne M (2015). Impact of a drug-free program on broiler chicken growth performances, gut health, *Clostridium perfringens* and *Campylobacter jejuni* occurrences at the farm level. Poult Sci.

[4] Van Immerseel F, Lyhs U, Pedersen K, Prescott J. Recent breakthroughs have unveiled the many knowledge gaps in *Clostridium perfringens*-associated necrotic enteritis in chickens: the first international conference on necrotic enteritis in poultry. Taylor & Francis; 2016. 10.1080/03079457.2016.1166857 27003036

[5] Rood JI, Adams V, Lacey J, Lyras D, McClane BA, Melville SB, Moore RJ, Popoff MR, Sarker MR, Songer JG (2018). Expansion of the *Clostridium perfringens* toxin-based typing scheme. Anaerobe.

[6] Keyburn AL, Boyce JD, Vaz P, Bannam TL, Ford ME, Parker D, Di Rubbo A, Rood JI, Moore RJ (2008). NetB, a new toxin that is associated with avian necrotic enteritis caused by *Clostridium perfringens*. PLoS Pathog.

[7] Gu C, Lillehoj HS, Sun Z, Lee Y, Zhao H, Xianyu Z, Yan X, Wang Y, Lin S, Liu L (2019). Characterization of virulent netB+/tpeL+ *Clostridium perfringens* strains from necrotic enteritis-affected broiler chicken farms. Avian Dis.

[8] Abildgaard L, Sondergaard TE, Engberg RM, Schramm A, Højberg O (2010). In vitro production of necrotic enteritis toxin B, NetB, by netB-positive and netB-negative *Clostridium perfringens* originating from healthy and diseased broiler chickens. Vet Microbiol.

[9] Cooper KK, Songer JG (2010). Virulence of *Clostridium perfringens* in an experimental model of poultry necrotic enteritis. Vet Microbiol.

[10] Li C, Lillehoj HS, Gadde UD, Ritter D, Oh S (2017). Characterization of *Clostridium perfringens* strains isolated from healthy and necrotic enteritis-afflicted broiler chickens. Avian Dis.

[11] Van Immerseel F, Rood JI, Moore RJ, Titball RW (2009). Rethinking our understanding of the pathogenesis of necrotic enteritis in chickens. Trends Microbiol.

[12] Coursodon C, Glock R, Moore K, Cooper K, Songer J (2012). TpeL-producing strains of *Clostridium perfringens* type A are highly virulent for broiler chicks. Anaerobe.

[13] Riddell C, Kong X-M (1992). The influence of diet on necrotic enteritis in broiler chickens. Avian Dis.

[14] Uzal FA, McClane BA, Cheung JK, Theoret J, Garcia JP, Moore RJ, Rood JI (2015). Animal models to study the pathogenesis of human and animal *Clostridium perfringens* infections. Vet Microbiol.

[15] Gholamiandehkordi AR, Timbermont L, Lanckriet A, Broeck WVD, Pedersen K, Dewulf J, Pasmans F, Haesebrouck F, Ducatelle R, Immerseel FV (2007). Quantification of gut lesions in a subclinical necrotic enteritis model. Avian Pathol.

[16] Stanley D, Wu S-B, Rodgers N, Swick RA, Moore RJ (2014). Differential responses of cecal microbiota to fishmeal, Eimeria and *Clostridium perfringens* in a necrotic enteritis challenge model in chickens. PLoS ONE.

[17] Sarmah H, Hazarika R, Tamuly S, Deka P, Manoharan S, Sharma RK (2021). Evaluation of different antigenic preparations against necrotic enteritis in broiler birds using a novel *Clostridium perfringens* type G strain. Anaerobe.

[18] Martin TG, Smyth JA (2009). Prevalence of netB among some clinical isolates of *Clostridium perfringens* from animals in the United States. Vet Microbiol.

[19] Shojadoost B, Vince AR, Prescott JF (2012). The successful experimental induction of necrotic enteritis in chickens by *Clostridium perfringens*: a critical review. Vet Res.

[20] Rood JI, Keyburn AL, Moore RJ (2016). NetB and necrotic enteritis: the hole movable story. Avian Pathol.

[21] Timbermont L, Lanckriet A, Gholamiandehkordi AR, Pasmans F, Martel A, Haesebrouck F, Ducatelle R, Van Immerseel F (2009). Origin of *Clostridium perfringens* isolates determines the ability to induce necrotic enteritis in broilers. Comp Immunol Microbiol Infect Dis.

[22] Keyburn AL, Yan X-X, Bannam TL, Van Immerseel F, Rood JI, Moore RJ (2010). Association between avian necrotic enteritis and *Clostridium perfringens* strains expressing NetB toxin. Vet Res.

[23] Smyth JA, Martin TG (2010). Disease producing capability of netB positive isolates of *C. perfringens* recovered from normal chickens and a cow, and netB positive and negative isolates from chickens with necrotic enteritis. Vet Microbiol.

[24] Immerseel FV, Buck JD, Pasmans F, Huyghebaert G, Haesebrouck F, Ducatelle R (2004). *Clostridium perfringens* in poultry: an emerging threat for animal and public health. Avian Pathol.

[25] Prescott J, Sivendra R, Barnum D (1978). The use of bacitracin in the prevention and treatment of experimentally-induced necrotic enteritis in the chicken. Can Vet J.

[26] Cooper K, Trinh H, Songer JG (2009). Immunization with recombinant alpha toxin partially protects broiler chicks against experimental challenge with *Clostridium perfringens*. Vet Microbiol.

[27] McReynolds J, Byrd J, Anderson R, Moore R, Edrington T, Genovese K, Poole T, Kubena L, Nisbet D (2004). Evaluation of immunosuppressants and dietary mechanisms in an experimental disease model for necrotic enteritis. Poult Sci.

